# Case Report: Atrial Fibrillation After Intravenous Administration of Iodinated Contrast Medium in a Patient With Hepatocellular Carcinoma

**DOI:** 10.1097/MD.0000000000001406

**Published:** 2015-09-04

**Authors:** Sergio Maimone, Roberto Filomia, Carlo Saitta, Giovanni Raimondo, Giovanni Squadrito

**Affiliations:** From Division of Clinical and Molecular Hepatology, University Hospital of Messina, Messina, Italy (SM, RF, CS, GR, GS); Department of Internal Medicine, University Hospital of Messina, Messina, Italy (GR); and Department of Human Pathology, University Hospital of Messina, Messina, Italy (GS).

## Abstract

We describe the case of a 73-year-old woman with liver cirrhosis and hepatocellular carcinoma (HCC) who developed 2 distinct episodes of paroxystic atrial fibrillation (AF) each of which occurred 1 to 4 hours after iodine medium contrast-enhanced computed tomography. Sinus rhythm was restored by amiodarone therapy after the first AF episode and by electrical cardioversion after the second one. A careful clinical, biochemical, and instrumental examination showed that the patient had subclinical hyperthyroidism and moderate mitral insufficiency with mild atrial enlargement.

Thus, the coexistence of both subclinical disthyroidism and of cardiac anatomical alterations may have predisposed the patient to AF that in fact occurred when exogenous iodine administration triggered a hyperthyroidism status.

## INTRODUCTION

Several iodine radioisotopes are used in medicine, either as scintigraphy tracers or as a part of contrast media in radiological procedures or as radiation therapy.^1^ The intake of exogenous sources of iodine may determine a condition of iodine-induced hyperthyroidism.^2^

This condition rarely occurs in individuals with a “normal” thyroid but it may affect patients with a variety of underlying misdiagnosed thyroid diseases (ie, Graves’ disease, multinodular goitre, subclinical hyperthyroidism, and iodine deficiency conditions).^3,4^ Hyperthyroidism is a form of thyrotoxicosis due to inappropriate increased synthesis and secretion of thyroid hormones.^1^ Indeed, thyrotoxicosis may have multiple etiologies and clinical manifestations, as well as different potential therapeutic treatments.^5^ A major complication of thyrotoxicosis is the worsening of a preexisting cardiac disease that may also lead to atrial fibrillation (AF), congestive heart failure, thromboembolism, and, rarely, death.^1,6^

Here, we report the case of a woman with liver cirrhosis and hepatocellular carcinoma (HCC) who suffered from 2 episodes of paroxystic AF occurring on 2 distinct occasions 1 to 4 hours after iodine contrast medium administration.

## CLINICAL CASE

This is the case of a 73-year-old woman affected by hepatitis B virus (HBV)-related cirrhosis. The HBV infection was diagnosed in 1981, when she underwent gastrectomy for complicated gastric ulcer. In February 1998, she underwent treatment with 3 million units of Interferon alpha 3 times per week per 24 weeks, without obtaining a biochemical and virological response. She was admitted to our Liver Unit in 1999. She appeared to be in satisfactory general clinical condition and reported being affected by osteoporosis (for which she was treated with calcium and cholecalciferol). She had no history of heart, lung, kidney, metabolic, or thyroid diseases. A needle liver biopsy was performed and histological cirrhosis was diagnosed. At that time the HBV-DNA quantification showed 330,000 copies/ml (COBAS AmpliPrep System, Roche Diagnostics, Monza, Italy) and therapy with 100 mg/die of lamivudine was therefore started. The treatment was promptly and persistently efficacious. Subsequently, the patient regularly attended the outpatient clinic of our liver unit. In February 2010, as a consequence of a slight increase of HBV-DNA levels (up to 300 IU/ml), tenofovir dipivoxil 245 mg/die therapy was introduced, replacing lamivudine.

**FIGURE 1:**
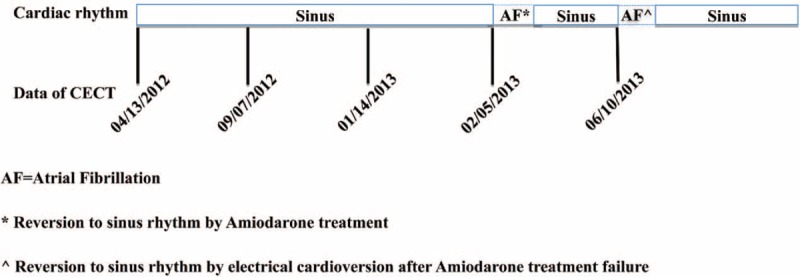
Representation of cardiac rhythm variations in a patient undergoing repeated contrast-enhanced computed tomography (CECT).

**FIGURE 2:**
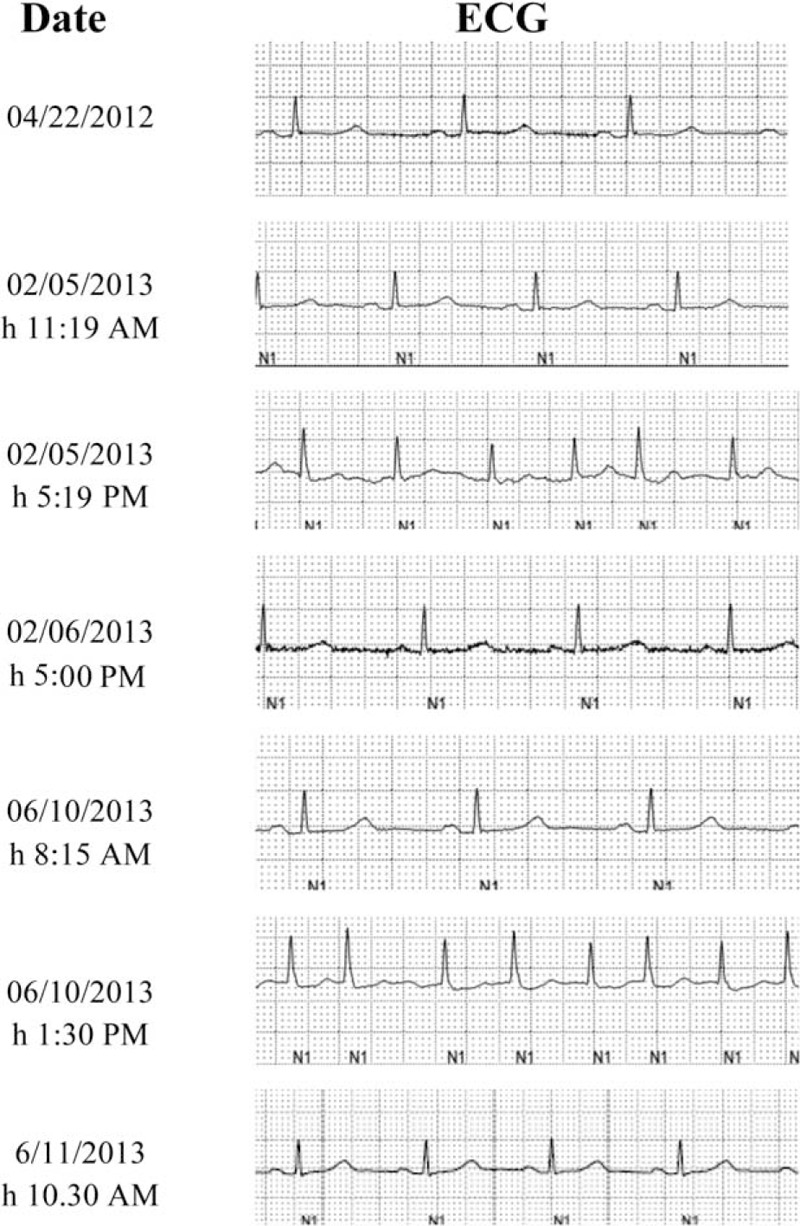
Electrocardiographic exams performed at different time points in a patient undergoing repeated CECT. 04/22/2012: ECG performed 10 months before the 1st episode of AF. 02/05/2013, 11.19 am: ECG performed before CECT. 02/05/2013, 05.19 pm: ECG performed 4 hours after CECT and showing AF. 02/06/2013, 05:00 pm: ECG performed after intravenous amiodarone administration and showing a reversion to sinus rhythm. 06/10/2013, 08.15 am: ECG performed before CECT. 06/10/2013, 1.30 pm: ECG performed 1 hour after CECT and showing AF. 6/11/2013, 10.30 am: ECG performed after electrical cardioversion and showing a reversion to sinus rhythm.

On November 3, 2011 the alpha-fetoprotein level rose to 217.5 ng/ml and an HCC nodule measuring 14 mm in diameter located in the right lobe was diagnosed by contrast iodine-enhanced computed tomography (CECT). Iodopride at a concentration of 370 mg/ml was the contrast medium used and it was administered intravenously at a dosage of 1.2 ml per kilogram of body weight. In December 2011, the patient underwent percutaneous radiofrequency ablation of the lesion and was subsequently followed up with periodic CECT examinations, according to AASLD practice guidelines^7^; a total of 3 CECTs were performed without the occurrence of any side effect. In occasion of the CECT control on February 5, 2013, the patient appeared to be in good general clinical condition, the blood pressure was 130/80 mm Hg, the electrocardiogram (ECG) was normal, the heart rate was 68 beats per minutes (bpm), the blood tests were normal, HBV-DNA was undetectable. However, 4 hours after the CECT scan with iodine contrast medium administration the patient complained of palpitations, dizziness, and epigastric pain. The blood pressure was 100/60 mm Hg, radial pulse was frequent and irregular, and ECG revealed the presence of high frequency AF (120 bpm). The cardiac enzymes, evaluated every 6 hours from the onset of AF for 24 hours, were persistently within normal ranges. She was treated intravenously with amiodarone 5 mg/kg for the first hour and subsequently at the dosage of 50 mg/hour for maintenance and with subcutaneous 6000 IU/die of enoxaparin. Twenty-four hours after onset of symptoms, there was a reversion to sinus rhythm and on February 6 the patient was discharged from the hospital (Figures 1 and 2). During hospitalization, an echocardiographic examination was performed which showed the presence of mitral annulus calcification with mitral insufficiency of moderate degree and mild enlargement of the left atrium.

Four months later the patient was scheduled for a further CECT scan control. At admission on June 10, she exhibited laboratory tests—including thyroid hormone values—within normal limits, she had no symptoms, her liver disease was compensated, the blood pressure was 110/60 mm Hg, the ECG revealed a sinus rhythm with heart rate of 68 bpm. One hour after the intravenous iodide contrast medium administration, she complained about an intense chest pain without any irradiation. The blood pressure was 100/70 mm Hg, her heart rate was irregular with 150 bpm and ECG analysis identified AF. The cardiac enzyme levels were within normal ranges. Therapy with aiodarone was promptly started but this time it had no clinical effect. Therefore the patient underwent electrical cardioversion, which succeeded in reverting to sinus rhythm (Figures 1 and 2). During hospitalization a neck ultrasonography examination was performed that showed an increased volume of the thyroid, many small hypo-echoic nodules (diameter <0.5 cm) and swollen cervical lymph nodes. Thyroid tests revealed a profile of subclinical hyperthyroidism [TSH 0.146 μIU/ml (normal range 0.3–4.2); FT3 0.9 ng/ml (normal range 0.87–1.78); FT4 9.12 μg/dl (normal range 6.09–12.23)]. On June 13, 2013 treatment with flecainide 100 mg/die was started; the patient showed no signs of AF and she was discharged from the hospital. Thereafter the patient underwent surveillance of HCC by both ultrasound and magnetic resonance examinations without showing recurrence of hepatic neoplasm so far. She did not experience any new episodes of AF (indeed, she is under flecanide treatment until today), hormone thyroid values remained within normal ranges and no further treatment for thyroid dysfunction was necessary.

## DISCUSSION

Iodine contrast media are the most common compounds used for enhancing X-ray-based imaging methods. Their administration is responsible for an excess of exogenous free iodine in the blood and may cause thyrotoxicosis in patients with preexisting thyroid abnormalities.^8^

Thyroid hormones have different mechanisms of action in different tissues, including vascular smooth muscle and myocardium.^9^ In the case of increased levels of thyroid hormones, an augmented contractile function of the myocardium has been observed, caused by upregulated synthesis of both myosin heavy chains and calcium ATPase.^10^ This hypercontractile state may lead to cardiac rhythm disturbances. Sinus tachycardia is the most common of such cardiac disturbances, but AF is also quite frequently observed since it occurs in 10% to 15% of patients.^11^

Although one cannot exclude that intravenous amiodarone therapy administered at the time of paroxysmal AF episodes might have contributed to thyroid dysfunction, the peculiar outcome observed in our patient can suggest that a cascade of events may have occurred depicting the following clinical scenario: a subclinical thyroid dysfunction as well as clinically silent mitral valve insufficiency and left atrium enlargement were chronically present; exogenous iodine administration triggered a hyperthyroid phase that in turn; led to AF favored by the preexisting cardiac anatomical abnormalities.

In conclusion, this peculiar and interesting case suggests the advisability of investigating the possible presence of subclinical forms of thyroid and cardiac alterations in patients requiring contrast iodine administration, above all when such procedures have to be repeatedly performed as in cirrhotic patients undergoing contrast enhanced tomography for the diagnostic and therapeutic management of HCC.
